# Indirubin derivative E804 inhibits angiogenesis

**DOI:** 10.1186/1471-2407-12-164

**Published:** 2012-05-03

**Authors:** Eun-Kyung Shin, Jin-Kyung Kim

**Affiliations:** 1 Center for Efficacy Assessment and Development of Functional Foods and Drugs, Hallym University, Chuncheon, Republic of Korea; 2 Department of Biomedical Science, Catholic University of Daegu, Daegu, Republic of Korea

**Keywords:** Angiogenesis, Indirubin derivative E804, Vascular endothelial growth factor receptor-2, Human umbilical vein endothelial cells, Tumor

## Abstract

**Background:**

It has previously been shown that indirubin derivative E804 (IDR-E804) blocks signal transducer and activator of transcription-3 signaling in human breast and prostate cancer cells and inhibits Src kinase activity. To further establish its role in angiogenesis, we tested its potential using human umbilical vein endothelial cells (HUVECs) and analyzed the effects of IDR-E804 on cellular and molecular events related to angiogenesis.

**Methods:**

The anti-angiogenic effects of IDR-E804 were examined by assessing the proliferation, migration and capillary tube formation of HUVECs were induced by vascular endothelial growth factor (VEGF) with or without various concentrations of IDR-E804. The inhibitory effect of IDR-E804 angiogenesis and tumor growth *in vivo* was also investigated in Balb/c mice subcutaneously transplanted with CT-26 colon cancer cells.

**Results:**

IDR-E804 significantly decreased proliferation, migration and tube formation of vascular endothelial growth factor VEGF-treated HUVECs. These effects were accompanied by decreased phosphorylation of VEGF receptor (VEGFR)-2, AKT and extracellular signal regulated kinase in VEGF-treated HUVECs. Intratumor injections of IDR-E804 inhibited the growth of subcutaneously inoculated CT-26 allografts in syngenic mice. Immunohistochemistry revealed a decreased CD31 microvessel density index and Ki-67 proliferative index, but an increased apoptosis index in IDR-E804-treated tumors.

**Conclusions:**

These data revealed that IDR-E804 is an inhibitor of angiogenesis and also provide evidence for the efficacy of IDR-E804 for anti-tumor therapies.

## Background

Angiogenesis has been described as one of the hallmarks of cancer, playing a fundamental role in tumor growth, invasion and metastasis [1]. Under many pathological conditions, including chronic inflammation, diabetic retinopathy, rheumatoid arthritis or atherosclerosis, persistent upregulated angiogenesis is a common feature [2,3]. Thus, understanding of the central importance of angiogenesis and how new blood vessels are formed has led to novel therapies designed to interrupt this process [2-4].

Angiogenesis is tightly controlled by balancing the activity of various angiogenic factors [2]. Numerous pathways contribute to tumor angiogenesis including vascular endothelial growth factor (VEGF), fibroblast growth factor, and platelet-derived growth factor [4]. Among these angiogenic factors, the signaling via VEGF is essential in the process of angiogenesis [5]. VEGF binds to two tyrosine kinase receptors, VEGF receptor (VEGFR)-1 and VEGFR-2 [4-6]. Signaling through VEGFR-1 and VEGFR-2 are important for embryonic development [5]. Although the affinity of VEGFR-2 for VEGF is lower than that of VEGFR-1, VEGFR-2 more potently stimulates endothelial cell proliferation and migration than VEGFR-1 [5,6]. Moreover, VEGFR-2 expression is almost entirely restricted to vascular endothelial cells and it has been reported that VEGFR-2 expression was markedly up-regulated during chronic inflammation, wound repair and tumor growth [5,6].

VEGF binding to the extracellular domain of VEGFR-2 results in dimerization and autophosphorylation of the intracellular tyrosine kinases [4-6]. This activates multiple downstream proteins, which play functional roles in cell survival, proliferation vascular permeability and stabilization of new blood vessels [4-6]. For example, VEGF induces endothelial cell proliferation by activating the protein kinase Ras-MEK-extracellular signal-regulated kinase (ERK) pathway [7]. The pro-survival effects of VEGF/VEGFR-2 are mediated by the PI3K/AKT pathway [7]. Accordingly, the VEGF signaling pathway has become an important target for anti-cancer treatment and many approaches have been developed to inhibit this pathway [8,9].

Indirubin has been shown to be the active component of the traditional Chinese herbal medicine, Danggui Longhui Wan, which is used to treat chronic myelogenous leukemia [10]. Various indirubin derivatives (IDRs) have been found to act as potent inhibitors of cyclin-dependent kinase (CDK)1/cyclin B, CDK2/cyclin A, CDK2/cyclin E, glycogen synthase kinase-3β and CDK5/p25, displaying potent growth inhibitory effects in several tumor cells [11-14]. Among the indirubin derivatives, IDR-E804 has been established as a strong inhibitor of signal transducer and activator of transcription (STAT)-3 signaling in human breast and prostate cancer cells [15]. In addition, IDR-E804 directly inhibits c-Src kinase activity *in vitro* and causes reduced phosphotyrosyl c-Src levels in human cancer cells [15]. Although the anti-cancer activity of IDR-E804 has been demonstrated in human breast and prostate cancer cells [15], the effect on angiogenesis, which is critical in cancer development, is still unknown.

Here, we report that IDR-E804 inhibited endothelial cell proliferation, migration and tube formation *in vitro* assays using human umbilical vein endothelial cells (HUVECs). In addition, IDR-E804 inhibited tumor growth *via* a reduction in CD31- and Ki-67-positive cells and increased apoptosis in the allograft colon tumor model. Furthermore, mechanistically, IDR-E804 directly inhibits VEGFR-2 kinase activity *in vitro* and causes a reduction of phosphorylation of VEGFR-2, AKT and ERK in VEGF stimulated HUVECs. Our studies suggest that IDR-E804 is a novel angiogenesis inhibitor and could be a potential drug candidate for angiogenesis related diseases.

## Methods

### Reagents

IDR-E804 was purchased from Calbiochem (Gibbstown, NJ). A 40 mM solution of IDR-E804 was prepared in dimethyl sulfoxide (Sigma-Aldrich, St. Louis, MO), stored at -20°C, and then diluted as needed with cell culture medium for *in vitro* experiments or with PBS for animal experiments. Recombinant human and mouse VEGF was obtained from eBioscience (San Diego, CA). Matrigel was purchased from BD Biosciences (San Jose, CA). The antibodies used in this study were anti-phospho-VEGFR-2 rabbit polyclonal, anti-VEGFR-2 rabbit polyclonal, anti-phospho-AKT rabbit polyclonal, anti-AKT rabbit polyclonal, anti-phospho-JNK rabbit polyclonal, anti-JNK, anti-phospho-pERK1/2 rabbit polyclonal, anti-ERK1/2 rabbit polyclonal (Cell Signaling Technology, Danvers, MA), and anti-β-actin mAb (Sigma-Aldrich).

### Cell line and proliferation assay

HUVECs were obtained from Lonza (Walkersville, MD) and cultured in EGM (Lonza) at 37°C in an atmosphere with 5% CO_2_. The effects of IDR-E804 on cell proliferation were tested using the CellTiter 96® AQ_ueous_ One Solution Cell Proliferation Assay (Promega, Madison, WI).

### Migration assay

HUVECs were allowed to grow to full confluence in 24-well plates that were precoated with 0.1% gelatin and then incubated with 10 μg/mL mitomycin C (Sigma-Aldrich) at 37°C in a 5% CO_2_ atmosphere for 2 h to inactivate HUVECs. Monolayer inactivated HUVECs were scratched by a 0.1 mL pipette tip. Fresh medium containing various concentrations of IDR-E804 was then added, and images were taken under the AxioImager M1 microscope (Carl Zeiss, Gottingen, Germany) after 8 h of incubation at 37°C.

### Tube formation assay

Matrigel was thawed at 4°C overnight, after which each well of prechilled 24-well plates was coated with 150 μL Matrigel and incubated at 37°C for 45 min. HUVECs (4 × 10^4^ cells) were then added in 1 mL EGM and incubated with the indicated amount of IDR-E804 at 37°C in a humidified 5% CO_2_ atmosphere. After 16 h of incubation, the medium was removed and rhodamine-labeled phalloidin (Thermo SCIENTIFIC, Rockford, IL) was added to stain the F-actin. Next, images of fluorescently labeled cells were collected using a ThermoScientific Cellomics ArrayScan High Content Screening Reader (Cellomics, Pittsburgh, PA) and analyzed by an automated algorithm that identified the tubes formed by the association and clustering of the endothelial cells [16].

### Aortic ring assay

Forty-eight-well plates were covered with 0.1 mL of Matrigel at 4°C and then incubated at 37°C under 5% CO_2_ for 30 min. Aortas isolated from SD rats (KOATECH, Pyeongtek, Korea) were cleaned of periadventitial fat and connective tissues, after which they were cut into 1-mm- to 1.5-mm-long rings. After being rinsed with PBS, the aortas were placed on the Matrigel-covered wells and covered with another 0.1 mL of Matrigel. Artery rings were cultured in 0.5 mL of EGM without serum for 24 h, after which the medium was replaced with 1.5 mL of EGM with vehicle or IDR-E804 (0.5, 1, 5 and 10 μM). The medium was changed every two days with fresh medium of the exact composition as described above. After seven days, the microvessel growth was measured by taking photographs with the AxioImager ZI inverted microscope (Carl Zeiss) using a 4x objective lens.

### VEGFR-2 inhibition assay

A 12.5 μL aliquot of the 4x reaction cocktail containing 100 ng VEGFR-2 [supplied from the HTScan VEGFR-2 kinase assay kit (Cell Signaling Technology)] was incubated with 12.5 μL of IDR-E804 for 5 min at room temperature. A 25 μL aliquot of 2x ATP/substrate peptide cocktail was then added to the preincubated reaction cocktail/IDR-E804 compound. After incubation at room temperature for 30 min, 50 μL of stop buffer (50 mM EDTA, pH 8) were added to each tube to stop the reaction. Next, 25 μL of each reaction were transferred into a 96-well streptavidin-coated plate containing 75 μL H_2_O/well and the samples were then incubated at room temperature for 60 min. After washing the wells three times with 200 μL/well PBS/T (0.05% Tween 20 in 1 × PBS), 100 μL of primary antibody [phosphorylated tyrosine monoclonal antibody (pTyr-100), 1:1000 in PBS/T with 1% bovine serum albumin (BSA)] were added per well. After being incubated at room temperature for 60 min, the wells were washed three times with 200 μL PBS/T, after which 100 μL of diluted HRP-labeled anti-mouse IgG (1:500 in PBS/T with 1% BSA) were added per well. Following incubation at room temperature for 30 min, the wells were washed five times with 200 μL of PBS/T per well. Subsequently, 100 μL of TMB substrate were added per well, and the plate was incubated at room temperature for 15 min. Stop solution (100 μL/well) was then added, and the samples were mixed and incubated at room temperature for 15 min. The plate was then read at 405 nm using a SpectraMax M2 microplate reader (Molecular Devices, Sunnyvale, CA).

### Western blot

HUVECs were treated with 0-10 μM IDR-E804 with or without human recombinant VEGF (10 ng/mL) for 30 min. Next, 10 μg of total cellular protein from each sample were subjected to western blotting with the indicated antibodies and immunoreactive proteins were detected using a chemiluminescence Western blotting detection system (ECL Plus^TM^ Western Blotting Reagents, Amersham Biosciences, Boston, MA).

### In vivo murine tumorigenesis assay

Five-week-old BALB/c male mice (KOATECH, Korea) weighing 20 g were divided into groups (5 mice/group). 5 × 10^5^ cells in 50 μl of PBS were mixed with 50 μl of matrigel and injected subcutaneously in the right hind flank of animals. Approximately five days after implantation when the tumors reached a volume of approximately 150 to 200 mm^3^, intratumor injections were given with 100 μL of vehicle (0.1% of DMSO in DW) or 200 μM (7.31 μg/animal) of IDR-E804 daily using a 26-gauge needle. Body weight and tumor volume were subsequently determined every two days by direct measurement with calipers (Mitutoyo Corporation, Japan). Tumor sizes were measured (tumor volume = ab^2^/2 in mm^3^, where a and b are the longest and the shortest perpendicular diameters of the tumor, respectively), and tumor weights were taken at termination on day 20. All animal studies were conducted under a protocol reviewed and approved by the Hallym University Institutional Animal Care and Use Committee.

### Immunohistochemistry

Tumors were removed 20 days after CT-26 cell injection and fixed with 4% paraformaldehyde for at least 24 h. The fixed tumors were embedded in paraffin, sectioned into 6-μm-thick sections, deparaffinized, and stained with hematoxylin and eosin. Apoptotic cells in the tumors were labeled by the terminal deoxyribonucleotidyl transferase-mediated dUTP-digoxigenin nick end-labeling (TUNEL) method using an In Situ Apoptosis Detection Kit (R&D Systems, Minneapolis, MN) according to the manufacturer’s instructions. Proliferating cells and platelet endothelial cell adhesion molecule-positive cells were assessed by the immunoperoxidase technique using an anti-Ki-67 antibody (Lab Vision, Fremont, CA) or anti-CD31 antibody (Santa Cruz Biotechnology, Santa Cruz, CA). Images were taken with an AxioImager M1 microscope (Carl Zeiss) and quantified by counting the number of positively stained cells in 15 randomly selected fields at x200 or x400 magnifications.

### Statistical analyses

The data are depicted as the means ± S.E. The values were evaluated by one-way analysis of variance (ANOVA) with Bonferroni multiple comparison post tests using the GraphPad Prism 4.0 software (GraphPad Software Inc., San Diego, CA). A *p*-value of less than 0.05 was considered to be statistically significant.

## Results

### IDR-E804 blocks angiogenesis in vitro

**Figure 1:**
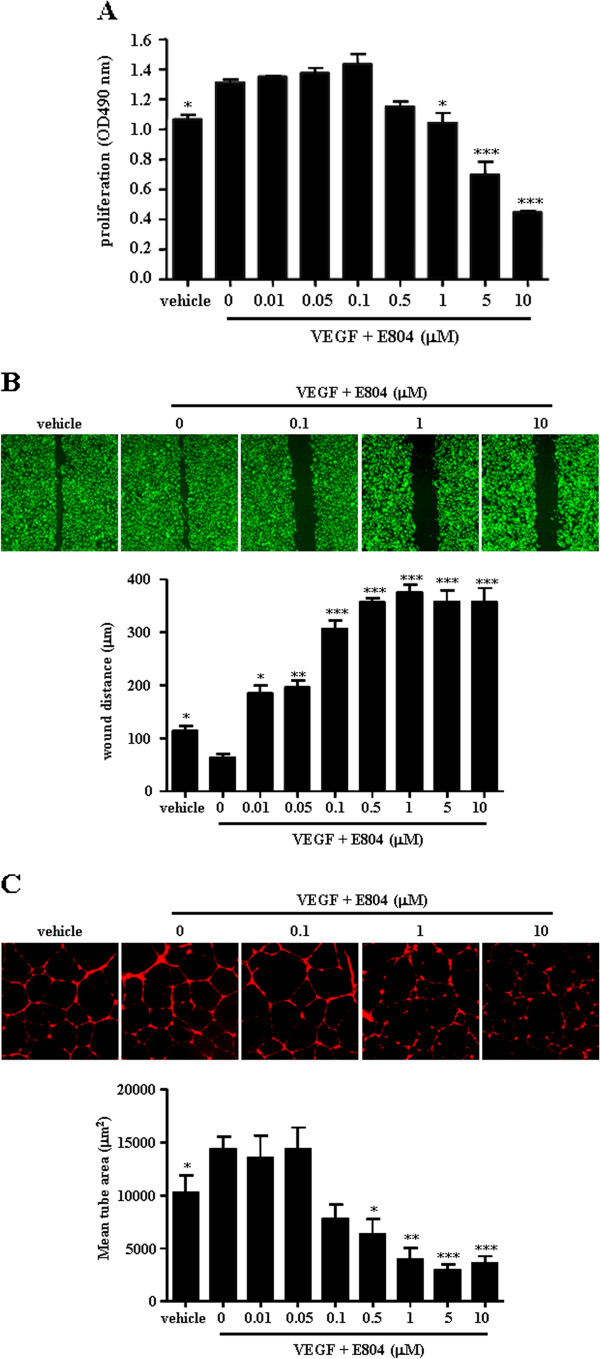
**Inhibition of endothelial cell proliferation, migration, and capillary-like tubule formation by IDR-E804.(A)** HUVECs were plated in 96-well plates, allowed to attach overnight, and then cultured for 24 h with vehicle or the indicated concentration of IDR-E804. The proliferation was measured as described in the Materials and Methods section. **(B)** For cell migration, a monolayer of inactivated HUVECs was scratched by a 0.1-mL pipette tip, and fresh medium containing vehicle or IDR-E804 was then added. After 24 h, migration of HUVECs was quantified. Original magnification, ×40. **(C)** For capillary-like tubule formation, HUVECs (4 × 10^4^/well) were seeded onto Matrigel-coated 24-well plates and incubated with vehicle or IDR-E804 at 37°C for 16 h. Endothelial tubules were then photographed and quantitated. Original magnification, ×40. The results shown are the means ± S.E. of four independent experiments conducted in triplicate. ^*^P < 0.05, ^**^P < 0.01, ^***^P < 0.001 versus VEGF-treated HUVECs.

Endothelial cell proliferation, migration and capillary tube formation are important events during angiogenesis. We first tested whether IDR-E804 inhibits proliferation in VEGF-treated HUVECs. Using an MTS assay, we measured HUVECs proliferation after treatment with various concentrations of IDR-E804 (0-10 μM). As shown in Figure 1A, IDR-E804 reduced cell proliferation in a concentration-dependent manner in VEGF-stimulated HUVECs.

We next measured the effect of IDR-E804 treatment on the migration of HUVEC cells using a scratch assays. To determine the extent of wound closure, monolayers of HUVECs were scratched with 0.1 mL tip and the initial distance was 450 ± 12 μm. In vehicle-treated controls, a large fraction of HUVECs migrated as shown in Figure 1B. IDR-E804 strongly inhibited the migration of HUVECs in a dose-dependent manner (Figure 1B).

When HUVECs are plated on a basement membrane matrix (Matrigel) in short-term culture, they align into networks of tubules (Figure 1C). This process is dependent on the proteolytic degradation of the matrix, cell realignment, and apoptosis; however, directed cell migration and proliferation are not involved in this process [17]. To evaluate tube formation by endothelial cells in a quantitative manner, tube length was measured using an imaging analyzer as previously described [16]. IDR-E804 reduced HUVEC tubule formation in a concentration-dependent manner (Figure 1C), with a significant reduction being observed at 0.5-10 μM. These data indicate that IDR-E804 has an anti-angiogenic effect in VEGF treated HUVECs.

### IDR-E804 inhibits microvessel outgrowth from the rat aortic ring

**Figure 2:**
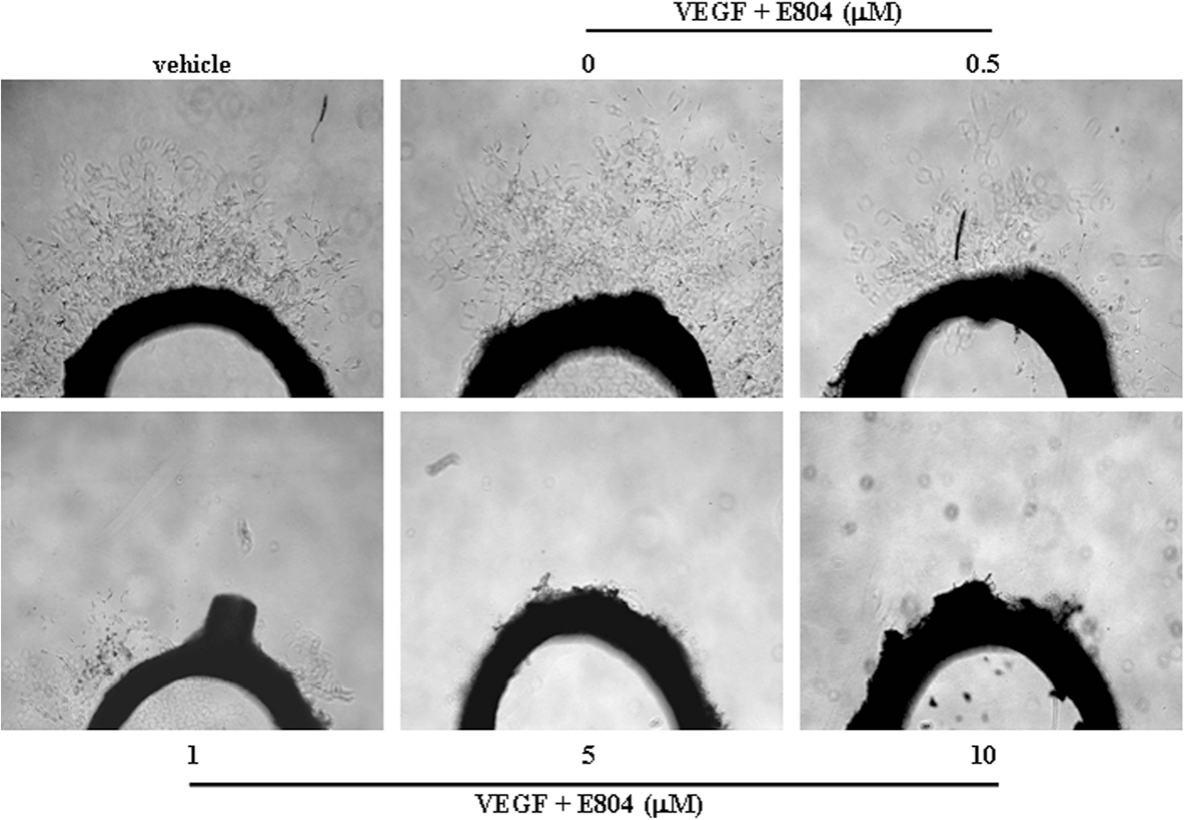
**Effect of IDR-E804 on microvessel outgrowth arising from rat aortic rings.** Aortic rings isolated from SD rats were embedded in Matrigel in 48-well plates and then fed with medium containing IDR-E804 for seven days. Photographs are representative of three independent experiments. Original magnification, ×40.

We next evaluated the anti-angiogenic effects of IDR-E804 in an *ex vivo* aorta sprout outgrowth assay. The 1-mm- to 1.5-mm-long aortic rings were placed on Matrigel and covered by another Matrigel layer and EGM with or without IDR-E804. After seven days of incubation, the numbers of microvessel outgrowths from the aortic rings in the presence or absence of IDR-E804 were compared. As shown in Figure 2, the presence of 0.5-10 μM IDR-E804 inhibited microvessel sprouting from the rat thoracic aorta, suggesting that IDR-E804 inhibited angiogenesis*.*

### IDR-E804 inhibits the VEGFR-2 signaling pathway and activity

**Figure 3:**
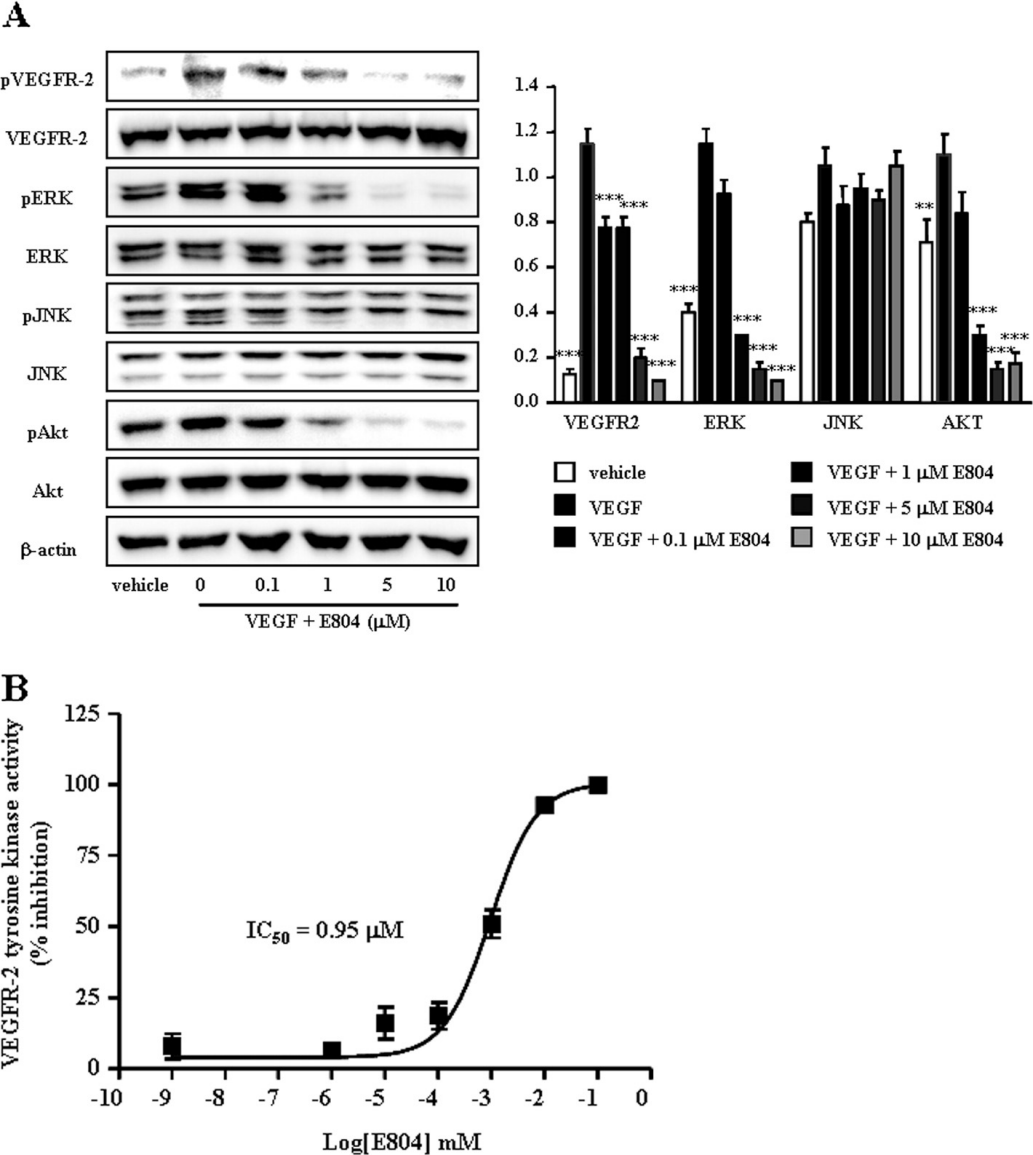
**Inhibition of VEGFR-2 signaling by IDR-E804.(A)** HUVECs were incubated with VEGF (10 ng/mL) and the indicated concentration of IDR-E804 for 30 min. Total cell lysates were prepared, proteins were separated by SDS–PAGE, and Western blot analysis was conducted. Photographs of chemiluminescent detection of the blots representative of four independent experiments are shown. Band intensity was quantified by densitometry and each bar represents the mean ± S.E. ^*^P < 0.05, ^**^P < 0.01, ^***^P < 0.001 versus VEGF-treated HUVECs. **(B)** Inhibition of VEGFR-2 activation by IDR-E804 in a specific VEGFR-2 inhibition assay. The results are reported as the mean ± S.E. of triplicate assays.

To determine if treatment with IDR-E804 inhibits the activation of VEGFR-2 and its multiple downstream signaling pathways, we next examined the levels of the phosphorylated-VEGFR-2, -ERK, -JNK and AKT proteins in IDR-E804 treated HUVECs. VEGFR-2 was phosphorylated by exogenous VEGF treatment in HUVECs as shown in Figure 3A, and IDR-E804 treatment reduced this phosphorylation. IDR-E804 also dose-dependently inhibited the phosphorylation of ERK and AKT proteins, a downstream signaling molecule, but not c-Jun N-terminal protein kinases, reaching almost basal levels at 1-10 μM (Figure 3A). The total steady state levels of VEGFR-2, AKT and ERK proteins remained unchanged, indicating that IDR-E804 specifically interferes with the phosphorylation of these proteins.

To verify the inhibitory effect of IDR-E804 on VEGFR-2, we examined the effects of various concentrations of IDR-E804 on the specific activation of VEGFR-2 using the HTScan® VEGFR-2 kinase assay kit according to the suggested protocol (Cell Signaling Technology). IDR-E804 inhibited VEGFR-2 kinase activity with an IC_50_ of 0.95 μM (Figure 3B), suggesting that E804 inhibits VEGFR-2 kinase activity.

### IDR-E804 inhibits tumor-angiogenesis and tumor growth in vivo

**Figure 4:**
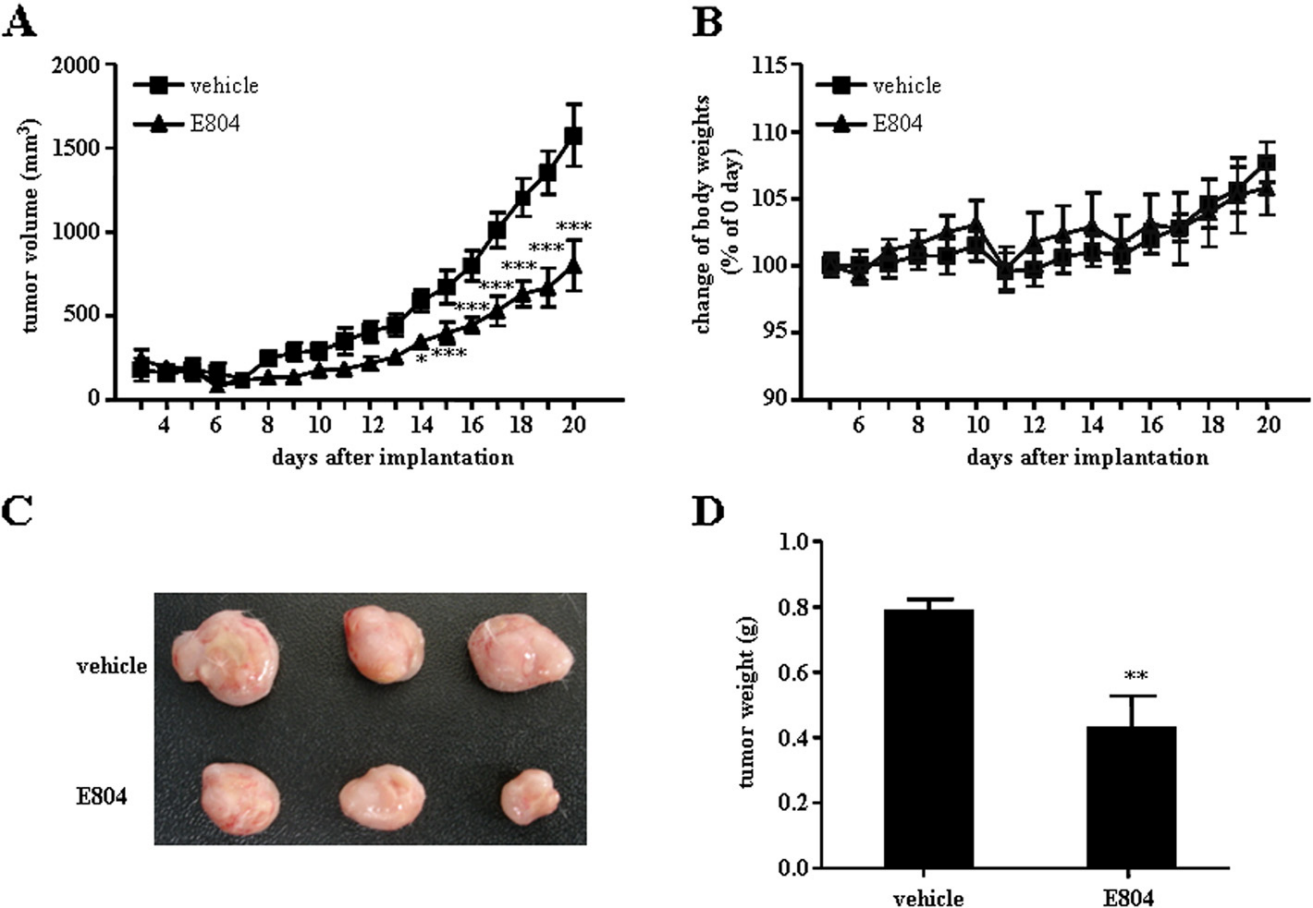
**IDR-E804 inhibits the growth of mouse tumors.** CT-26 cells were injected subcutaneously into the right flank. After tumor formation, mice were treated with vehicle or IDR-E804 and scarified at day 20 after injection of tumor cells for the analysis. **(A)** Tumor growth after IDR-E804 treatment. Tumor sizes were measured daily with a caliper. **(B)** Changes in the body weight of mice treated with vehicle or IDR-E804. **(C)** Macroscopic features of tumors isolated from vehicle or IDR-E804 treated mice. **(D)** Tumor weight of vehicle or IDR-E804 treated mice. The results shown are the means ± S.E. of two independent experiments conducted. ^*^P < 0.05, ^**^P < 0.01, ^***^P < 0.001 versus vehicle treated animals.

Tumor angiogenesis presents nutrients, oxygen, and critical routes for tumor growth as well as metastasis [18]. The *in vivo* anti-angiogenic and anti-tumor activity of IDR-E804 was assessed using murine colon carcinoma CT-26 cells inoculated subcutaneously into syngeneic BALB/c mice. As shown in Figure 4B, IDR-E804 daily treatment (total 15 times) did not alter the body weight, but the average tumor size of the control group was 1576 ± 260 mm^3^ while that of the IDR-E804 treated group was 798 ± 212 mm^3^ at day 20 after injection of tumor cells (Figure 4A). The average tumor weight of the control group was 0.79 ± 0.03 g, while that of the IDR-E804 treated group was 0.42 ± 0.09 g (Figure 4D) at day 20 after injection of tumor cells, indicating that IDR-E804 significantly inhibited tumor growth.

**Figure 5:**
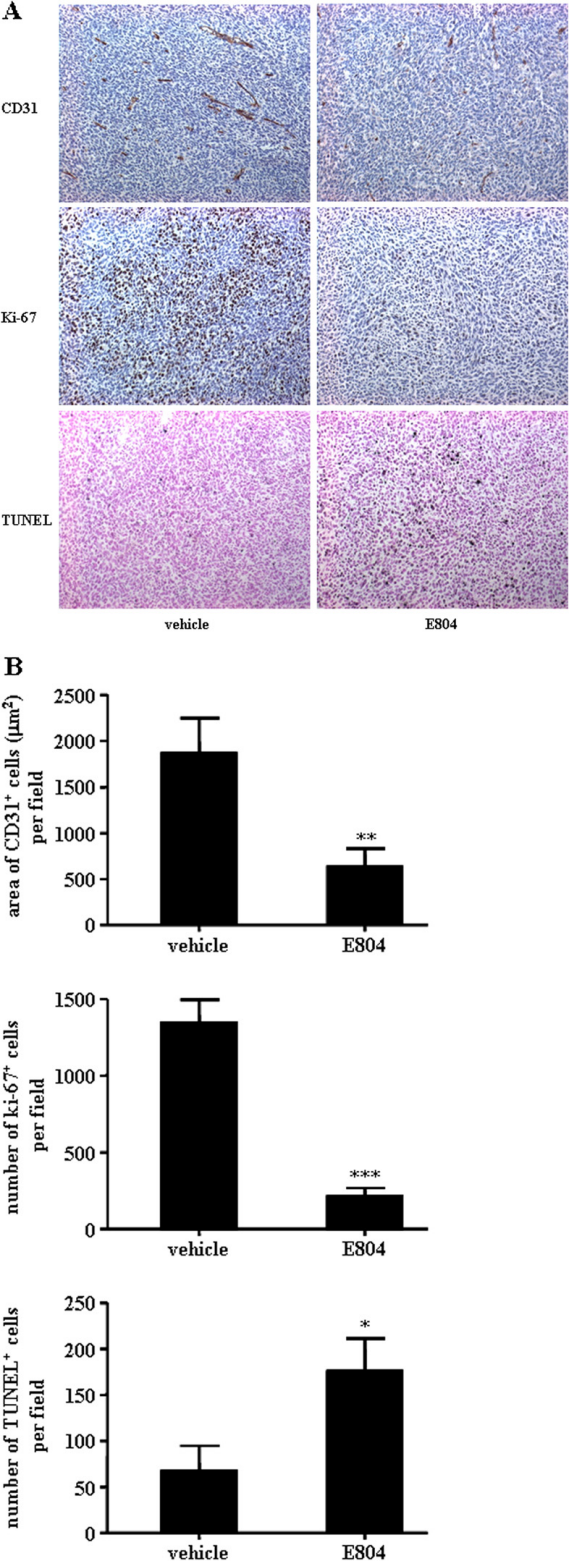
**IDR-E804 inhibits angiogenesis and cell proliferation and induces apoptosis in mouse tumors.(A)** Immunohistochemistry was performed in tumor tissues derived from vehicle and IDR-E804 treated mice on day 20 to measure angiogenesis by CD31 staining, cell proliferation by Ki-67 staining and apoptosis by TUNEL assay. Original magnification, ×200. **(B)** Quantitation of CD31 vessel staining, Ki-67 staining, and TUNEL staining from immunohistochemical analyses are shown. Results are the mean ± S.E. of 20 sections per group. ^*^P < 0.05, ^**^P < 0.01, ^***^P < 0.001 versus vehicle-treated animals.

To examine the effect of IDR-E804 on tumor angiogenesis, we stained the tumor sections with the specific endothelial cell marker, CD31 protein. Vehicle-treated mice showed 1863 ± 379 μm^2^ CD31-immunoreactive areas per field, whereas IDR-E804-treated mice showed 703 ± 102 μm^2^ per field (Figure 5), indicating that IDR-E804 significantly inhibited tumor angiogenesis and thereby prevented tumor growth.

We next tested whether IDR-E804 causes histological changes in tumor tissue by measuring proliferation and apoptosis using Ki-67 and TUNEL staining, respectively. In similar fields of view, IDR-E804 mice showed fewer proliferative cells and more apoptotic cells than control mice (Figure 5). Collectively, these data suggest that IDR-E804 inhibits tumor angiogenesis, and subsequently promotes apoptosis and reduces tumor progression.

## Discussion

IDR-E804 is a cell-permeable indirubin derivative that blocks the STAT-3 signaling pathway [15]. Previous studies have demonstrated that IDR-E804 is a promising anti-cancer agent because it is able to inhibit the proliferation and induce the apoptosis of various human cancer cells [15]. IDR-E804 has also been shown to be a potent, reversible, and ATP-competitive inhibitor of the kinase activities of Src, Cdk1/cyclin E, Cdk2/cyclin A, and Cdk1/cyclin [15]. This compound has also been shown to reduce the phosphorylation levels of Src, JAK1, and STAT-3 in MDA-MB-468 human breast cancer cells [15]. In addition to the inhibitory activities of Src, Cdk1/cyclin E, Cdk2/cyclin A, and Cdk1/cyclin, the apoptotic effect of IDR-E804 has been shown to occur in response to the down-regulation of anti-apoptotic proteins Mcl-1 and survivin. These anti-cancer effects of IDR-E804 in various human cancer cells led us to investigate the role of IDR-E804 in angiogenesis, which is essential for cancer development [1-3].

The present study provides evidence that IDR-E804 is a VEGFR-2 inhibitor that inhibits angiogenesis and tumor progression. Our work focused on the inhibitory effects of IDR-E804 on the proliferation, migration and tube formation in HUVECs, which are critical steps involved in endothelial angiogenesis. Due to the inhibition of VEGFR-2 phosphorylation and activation, IDR-E804 reduced the ERK and AKT signaling pathway in HUVECs (Figure 3). We also found that IDR-E804 directly inhibited the kinase activity of purified VEGFR-2, a novel activity of IDR-E804 that has not yet been characterized. To the best of our knowledge, this is the first study to demonstrate the inhibitory effect of IDR-E804 on angiogenesis via inhibition of VEGF/VEGFR-2 signaling, at least in part. We also demonstrated that IDR-E804 effectively suppresses the growth of CT-26 colorectal cancer grafts via inhibition of angiogenesis as well as acceleration of apoptosis (Figures 4 and 5). Since previous results indicated that IDR-E804 inhibits Src kinase activity and STAT-3 phosphorylation in various cancers which are critical for angiogensis [19,20], it is possible that IDR-E804 inhibits angiogenic process via Src and/or STAT-3 signaling pathways, but the detail action of IDR-E804 on Src and STAT-3 activation in HUVECs requires further investigation.

Our *in vitro* studies with HUVECs demonstrated that IDR-E804 inhibited the proliferation, migration and capillary-like structure formation in VEGF-stimulated HUVECs (Figure 1). A similar phenomenon was observed in the rat aortic ring assay (Figure 3), suggesting that IDR-E804 inhibits microvessels formation. Endothelial cell signaling in response to VEGF is well established [4-7]. VEGF is one of the most potent tumor angiogenic factors that promotes the proliferation and migration of endothelial cells and increases vascular permeability [5-8]. It is well known that VEGF and its receptors are important mediators during different steps of angiogenesis in cancer [21]. For its multifunction, VEGF activates a diverse and integrated network of signaling pathways. These different signaling cascades play different roles in the biological functions and also cross-talk to each other. The survival effect of VEGF activation is primarily mediated by the AKT pathway. AKT not only inhibits the pro-apoptotic proteins and apoptotic caspases, but also up-regulates the anti-apoptotic proteins [22]. ERK signaling has been implicated in the proliferation, survival, and protection against receptor-mediated apoptosis of endothelial cells [23]. Moreover, ERK signaling is thought to stimulate angiogenesis by promoting endothelial cell motility [24]. In the present study, the phosphorylation of the AKT and ERK of HUVECs was increased upon VEGF stimulation and IDR-E804 significantly reduced AKT and ERK phosphorylation. These results indicate that E804 inhibits proliferation and migration of endothelial cells *via* inhibition of AKT and ERK.

The present study indicates that IDR-E804 (20 μM) effectively suppressed tumor volume and tumor weight (Figure 4A and 4D) without adverse effects on mouse body weight (Figure 4B) *via* inhibition of angiogenesis as well as acceleration of apoptosis in allograft colon tumor mice (Figure 5). Previous studies demonstrated that IDR-E804 induced apoptosis in transformed MDA-MB-468 human breast cancer cells, but did not induce apoptosis in normal MCF-10A cells. In addition, Mcl-1 and survivin expression is dramatically reduced in response to IDR-E804 and apoptosis is induced in breast cancer cells [15]. Our immunohistochemistry results revealed that the generation of new blood vessels and Ki-67 positive cells in the treated group were reduced when compared with the control groups (Figure 5). Moreover, the numbers of TUNEL positive cell were significantly higher in IDR-E804 treated tumor sections (Figure 5). These findings indicate that IDR-E804 induced the apoptosis in cancer cells as well as tumor tissues in mice.

Previous studies demonstrated that indirubin (C_16_H_10_N_2_O_2_) and indirubin-3’-monoxime (C_16_H_11_N_3_O_2_), an indirubin derivative, are potential drug candidates for angiogenesis related diseases [25,26]. Indirubin inhibited prostate tumor growth mainly through antitumor angiogenesis via blocking VEGFR-2 mediated STAT-3 signaling pathway in endothelial cell [26]. In addition, indirubin-3’-monoxime also blocked the proliferation, migration, and capillary-like structure formation of HUVECs [25]. Comparing previous study, IDR-E804 exerted anti-angiogencic activity in HUVECs at lower concentration than indirubin and indirubin-3’-monoxime. The inhibited concentration of indirubin and indirubin-3’-monoxime against the proliferation, migration as well as tube formation of HUVECs was 25-100 μM and 2.5-20 μM, respectively [25,26]. Although indirubin blocked the phosphorylation of VEGFR-2, the concentration which blocks the phosphorylation of VEGFR-2 was 25-100 μM whereas IDR-E804 was 1-10 μM. Although additional work is needed to elucidate the relationship between the structure and anti-angiogenic activity of indirubin or other indirubin derivatives, IDR-E804 might have powerful potential to inhibit angiogenesis than indirubin and indirubin-3’-monoxime.

It should be noted that this *in vivo* study has a few limitations. Since we tested only a single dose of IDR-E804 for the *in vivo* mouse experiments, detailed dose-response effect of IDR-E804 on angiogenesis and tumor growth *in vivo* needs to be investigated. In addition, preclinical studies to test the safety of IDR-E804 *in vivo* should be conducted to determine if it is suitable for further use as an anti-cancer and anti-angiogenic agent.

## Conclusion

In summary, our studies show that IDR-E804 functions as an inhibitor of the VEGFR-2 signaling pathway, leading to inhibition of angiogenesis. Our data suggest a new mechanism of action for IDR-E804 and its potential use as an anti-angiogenic and anti-cancer agent.

## Competing interests

The authors declare no conflict of interest for this article.

## Authors’ contributions

Conception and design: J-KK, *In vitro* and *in vivo* studies: E-KS, Manuscript writing: J-KK and E-KS. Final approval of manuscript: J-KK. Both authors have read and approved the final manuscript.

## Pre-publication history

The pre-publication history for this paper can be accessed here:

http://www.biomedcentral.com/1471-2407/12/164/prepub
